# First Detection of *Theileria sinensis*-like and *Anaplasma capra* in *Ixodes kashmiricus*: With Notes on *cox1*-Based Phylogenetic Position and New Locality Records

**DOI:** 10.3390/ani13203232

**Published:** 2023-10-17

**Authors:** Muhammad Numan, Abdulaziz Alouffi, Mashal M. Almutairi, Tetsuya Tanaka, Haroon Ahmed, Haroon Akbar, Muhammad Imran Rashid, Kun-Hsien Tsai, Abid Ali

**Affiliations:** 1 Department of Zoology, Abdul Wali Khan University Mardan, Mardan 23200, Pakistan; 2 King Abdulaziz City for Science and Technology, Riyadh 12354, Saudi Arabia; 3 Department of Pharmacology and Toxicology, College of Pharmacy, King Saud University, Riyadh 11451, Saudi Arabia; 4 Laboratory of Infectious Diseases, Joint Faculty of Veterinary Medicine, Kagoshima University, Kagoshima 890-0065, Japan; k6199431@kadai.jp; 5 Department of Biosciences, COMSATS University Islamabad (CUI), Park Road, Chak ShahZad, Islamabad 45550, Pakistan; 6 Department of Parasitology, University of Veterinary and Animal Sciences, Lahore 54200, Pakistan; 7 Global Health Program, Institute of Environmental and Occupational Health Sciences, College of Public Health, National Taiwan University, Taipei 106319, Taiwan

**Keywords:** *Ixodes kashmiricus*, *cox1*, *Theileria sinensis*-like, *Anaplasma capra*, transhumant herds, Pakistan

## Abstract

**Simple Summary:**

*Ixodes* species are the main vectors of bacteria and piroplasm for different vertebrate hosts. Research on these unexplored concerns has been neglected in different regions including Pakistan. Recently, we molecularly characterized *Ixodes kashmiricus* ticks and associated *Rickettsia* spp.; however, the *cox1* sequence and associated *Theileria* spp. and *Anaplasma* spp. for this tick are unknown. This study aimed to genetically identify *I. kashmiricus* based on the *cox1* sequence and associated *Theileria* spp. and *Anaplasma* spp. A total of 352 ticks including adult females, nymphs and males were collected from small ruminants. The BLAST results and phylogenetic analysis of the *cox1* sequence revealed a close resemblance with the *Ixodes ricinus* complex sequences. The 18S rDNA and 16S rDNA sequences showed maximum identity with *Theileria* cf. *sinensis* or *Theileria sinensis* and *Anaplasma capra*, respectively, and they phylogenetically clustered with the same species. This is the first report on the *cox1* sequence of the *I. kashmiricus* tick, new locality records, and associated *T. sinensis*-like and *A. capra*. In order to determine the epidemiology of *Ixodes* ticks and their related pathogens, a widespread tick investigation is required.

**Abstract:**

*Ixodes* ticks transmit *Theileria* and *Anaplasma* species to a wide range of animals. The spreading of ticks and tick-borne pathogens has been attributed to transhumant herds, and research on these uninvestigated issues has been neglected in many countries, including Pakistan. Recently, we used internal transcribed spacer (ITS) and 16S ribosomal DNA partial sequences to genetically characterize *Ixodes kashmiricus* ticks and their associated *Rickettsia* spp. However, the data on its *cox1* sequence and associated *Theileria* spp. and *Anaplasma* spp. are missing. This study aimed to genetically characterize *I. kashmiricus* based on the *cox1* sequence and their associated *Theileria* spp. and *Anaplasma* spp. The *I. kashmiricus* ticks were collected from small ruminants: sheep (*Ovis aries*) and goats (*Capra hircus*) of transhumant herds in district Shangla, Dir Upper and Chitral, Khyber Pakhtunkhwa (KP), Pakistan. Out of 129 examined hosts, 94 (72.87%) (56 sheep and 38 goats) were infested by 352 ticks, including adult females (175; 49.7%) followed by nymphs (115; 32.7%) and males (62; 17.6%). For molecular analyses, 121 ticks were subjected to DNA isolation and PCR for the amplification of the *cox1* sequence for *I. kashmiricus*, 18S rDNA for *Theileria* spp. and 16S rDNA sequences for *Anaplasma* spp. The obtained *cox1* sequence showed 89.29%, 88.78%, and 88.71% identity with *Ixodes scapularis, Ixodes gibbosus,* and *Ixodes apronophorus*, respectively. Phylogenetically, the present *cox1* sequence clustered with the *Ixodes ricinus* complex. Additionally, the 18S rDNA sequence showed 98.11% maximum identity with *Theileria* cf. *sinensis* and 97.99% identity with *Theileria sinensis*. Phylogenetically, *Theileria* spp. clustered with the *T.* cf. *sinensis* and *T. sinensis*. In the case of *Anaplasma* spp., the 16S rDNA sequence showed 100% identity with *Anaplasma capra* and phylogenetically clustered with the *A. capra*. PCR-based DNA detection targeting the amplification of *groEL* and *flaB* sequences of *Coxiella* spp. and *Borrelia* spp., respectively, was unsuccessful. This is the first phylogenetic report based on *cox1* and new locality records of *I. kashmiricus*, and the associated *T. sinensis*-like and *A. capra*. Significant tick surveillance studies are needed in order to determine the epidemiology of *Ixodes* ticks and their associated pathogens.

## 1. Introduction

Genus *Ixodes* (Acari: Ixodidae: Prostriata) developed during the Mesozoic era’s cretaceous period (65–95 million years ago) [1,2]. The *Ixodes* genus comprises more than 265 species, which are divided based on morphology into 18 subgenera [3]. Among them, the largest subgenus *Ixodes* comprises 18 species and includes the most studied ticks [4]. *Ixodes* ticks are known to adopt in particular environmental conditions for survival and development, and these are considered to limit their dispersal [3,5]. Climatic conditions and the availability of a suitable host are the two most important factors determining the distribution and abundance of *Ixodes* ticks. *Ixodes* ticks have been commonly found in woodland or mixed forest and grassland, which provide moist vegetation and approximately 80% humidity—a critical threshold for the survival and development of these ticks [2,5].

*Ixodes* ticks are known to parasitize a wide range of hosts including birds, reptiles, and mammals [3]. These ticks are capable of transmitting pathogens of medical and veterinary importance like *Theileria* spp., *Anaplasma* spp., *Coxiella* spp., *Babesia* spp. and *Borrelia* spp. [5,6,7,8]. Hard ticks, particularly of the *Haemaphysalis*, *Dermacentor*, *Ixodes* and *Rhipicephalus* genera are the primary vectors that transmit *Anaplasma* spp. [9,10]. To date, only two species of *Anaplasma* spp. like *Anaplasma phagocytophilum* have been detected in *Ixodes* ticks such as *Ixodes ricinus* [11], *Ixodes trianguliceps* [12], *Ixodes scapularis* [13] and *Ixodes frontalis* [14], while the *Anaplasma capra* has been detected in *Ixodes persulcatus* [15]. Several other hard tick species, including *Haemaphysalis longicornis*, *Haemaphysalis qinghaiensis, Rhipicephalus sanguineus, Rhipicephalus turinicus, Rhipicephalus haemaphysaloides, Rhipicephalus microplus* and *Dermacentor everstianus* have been shown as carrier of *A. capra* [9,10,16]. Similarly, some piroplasm species such as *Theileria annae* in *Ixodes hexagonus* [17], *Theileria fuliginosa* in *Ixodes australiensis* [18], and *Theileria* spp. in *I. ricinus* [14] have been described. On the other hand, *Ha. qinghaiensis* is the only known vector of *Theileria sinensis* [19]. *Coxiella* spp. such as *Coxiella burnetii* [7], and *Borrelia* spp. such as *Borrelia burgdorferi*, *Borrelia miyamotoi*, *Borrelia genospecies* and “*Candidatus* Borrelia sibirica” of the relapsing fever group, have been detected in the *Ixodes* ticks [6,20].

The identification of ticks, particularly those belonging to the genus *Ixodes*, has been traditionally based on morphological features, such as the shape of the spiracular plates, grooves of the scutum and punctations [2,4,21]. However, these methods are often insufficient for accurate identification and differentiation, particularly for *Ixodes* and other closely related ticks [22,23]. Molecular techniques have been alternatively used for the accurate identification and differentiation of different tick species [22,24,25,26,27,28]. Some genetic markers, such as *cox1*, 16S ribosomal DNA (rDNA) and internal transcribed spacer (ITS), have been shown suitable for the accurate delineation of ticks [21,29,30,31,32]. *Ixodes kashmiricus* tick has been reported based on ITS and 16S rDNA sequences, and their associated *Rickettsia* spp. has been reported based on *gltA* and *ompA* sequences [21]. However, genetic data based on *cox1* sequence for *I. kashmiricus* and associated *Theileria* spp. and *Anaplasma* spp. are missing. Herein, *I. kashmiricus* ticks were for the first time genetically characterized based on a mitochondrial *cox1* sequence and screened for associated *Theileria* spp. and *Anaplasma* spp. in Khyber Pakhtunkhwa (KP), Pakistan.

## 2. Materials and Methods

### 2.1. Ethical Approval

This study was approved by the Advance Studies Research Board (ASRB: Dir/A&R/AWKUM/2022/9396) committee members of Abdul Wali Khan University, Mardan KP, Pakistan. The oral permission was obtained from the owners of the transhumant herds during the host’s observation and tick collection.

### 2.2. Study Area and Tick Collection

**Figure 1:**
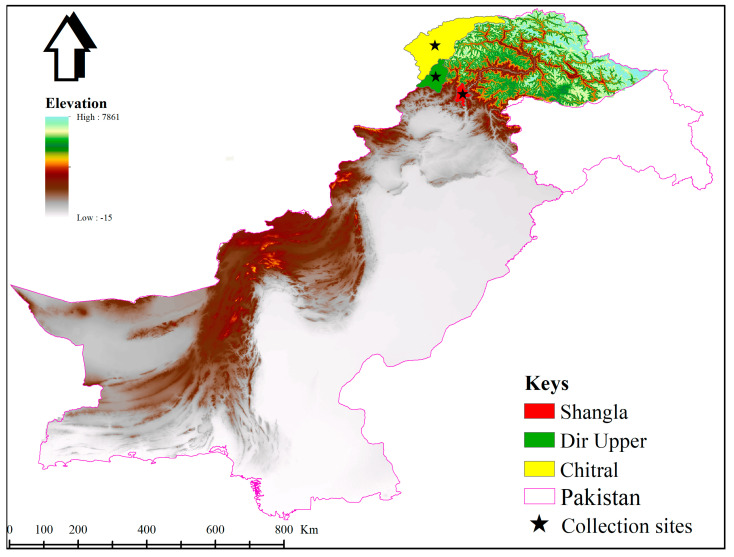
Map showing the locations (black stars) where *Ixodes* ticks were collected during this study.

This study was conducted in district Shangla (34°46′34.6″ N 72°40′45.8″ E), Dir Upper (35°13′23.4″ N 71°55′12.2″ E) and Chitral (35°50′11.7″ N 71°48′18.0″ E) of KP, Pakistan. These districts are highly mountainous, with an elevation approximately 3000–3500 m (m), and situated in the north or northwest of KP. The elevation study map was designed in ArcGIS 10.3.1, using the “Global Positioning System” to determine the locations of the collection sites (Figure 1). Tick specimens were collected from small ruminants in transhumant herds during May–July 2022 in district Shangla, Dir Upper and Chitral. The ticks were isolated from the host body carefully via tweezers to avoid any external damage to the specimens. The tick specimens were washed in distilled water followed by 70% ethanol and preserved in 100% ethanol in 1.5 mL Eppendorf tubes for further experiments.

### 2.3. Morphological Identification of Ticks

The collected tick specimens were morphologically identified under a stereozoom microscope (StereoBlue-euromex SB.1302-1, Arnhem, The Netherlands) using standard morphological identification keys [2,21,33].

### 2.4. DNA Isolation and PCR

Individually, 121 ticks including 20 males, 44 adult females and 27 nymphs from sheep, as well as 16 females and 14 nymphs from goats, were selected and subjected to molecular analyses. Before the DNA isolation, tick specimens were washed with distilled water followed by 70% ethanol and kept in an incubator (30–45 min) until dried. The specimens were cut with sterile scissors and homogenized in 200–300 µL phosphate-buffered saline (pH 7.4, 137 mM NaCl, 2.7 mM KCl, 8 mM Na_2_HPO_4_, 2 mM KH_2_PO_4_) using a micro-pestle. The genomic DNA was extracted using a phenol–chloroform protocol [34], and the isolated DNA pellet was diluted by the addition of 20–30 μL of “nuclease-free” PCR water. The isolated genomic DNA was quantified via NanoDrop (Nano-Q, Optizen, Daejeon, Republic of Korea) and stored at −20 ℃.

The tick genomic DNA of *I. kashmiricus* (1 male, 2 adult females, and 2 nymphs) were subjected to conventional PCR (GE-96G, BIOER, Hangzhou, China) for the amplification of mitochondrial cytochrome C oxidase 1 (*cox1*) sequence. Each PCR reaction mixture contained 25 µL volume—comprising 1 µL of each primer (10 µM), 2 µL of template DNA (50–100 ng/µL), 8.5 µL of PCR water “nuclease-free” and 12.5 µL of Dream*Taq* green MasterMix (2×) (Thermo Scientific, Waltham, MA, USA).

All extracted genomic DNA was used for the screening of associated pathogens based on genetic markers such as 18S rRNA for *Theileria* spp., 16S rRNA for *Anaplasma* spp., *groEL* for *Coxiella* spp. and *flaB* for *Borrelia* spp. Each PCR contained a positive control (DNA of *Anaplasma marginale*, *Theileria annulata, Coxiella burnetii* and *Borrelia anserina* for pathogens and genomic DNA of *Hy. anatolicum* for ticks) and a negative control (“nuclease-free” PCR water instead of DNA). The primers used in this study and their thermocycler conditions are given in Table 1.

The PCR-amplified products were electrophoresed on a 1.5% agarose gel and visualized under ultraviolet light in the Gel Documentation System (BioDoc-It™ Imaging Systems UVP, LLC, Upland, CA, USA). PCR-positive samples were purified by using a DNA Clean & Concentrator Kit (Zymo Research, Irvine, CA, USA) by following the manufacturer’s instructions.

### 2.5. DNA Sequencing and Phylogenetic Analysis

All amplified amplicons of *cox1* (5: 1 male, 2 adult females, and 2 nymphs) for ticks, 18S rDNA (2: 1 adult female and 1 nymph) for *Theileria* spp. and 16S rDNA (4: 2 adult females and 2 nymphs) for *Anaplasma* spp. were sequenced (Macrogen Inc., Seoul, Republic of Korea) by Sanger sequencing. The obtained sequences were trimmed/edited via SeqMan v. 5 (DNASTAR, Inc., Madison/WI, USA) for the removal of poor reading sequences and subjected to Basic Local Alignment Search Tool (BLAST, https://blast.ncbi.nlm.nih.gov/Blast.cgi, accessed on: 10 July 2022) at the National Center for Biotechnology Information (NCBI, https://www.ncbi.nlm.nih.gov/, accessed on: 10 July 2022). After BLAST, maximum identity sequences of the most similar/subgenus species were downloaded in FASTA format from the NCBI. Obtained sequences were aligned with the downloaded sequences using ClustalW multiple alignments in BioEdit Sequence Alignment Editor v. 7.0.5 [39]. The phylogenies were constructed individually for each DNA sequence of tick and associated pathogens through the Maximum Likelihood statistical method and Kimura 2-parameter model in Molecular Evolutionary Genetics Analysis (MEGA-X) with a bootstrapping value of 1000 [40]. The coding sequences like *cox1* sequences were aligned by using MUSCLE algorithms [41].

### 2.6. Statistical Analyses

All recorded data such as the numbers of collected ticks and their life stages in the three districts, as well as associated pathogens like *Theileria* spp. and *Anaplasma* spp., were arranged in the spreadsheet (Microsoft Excel v. 2016, Microsoft 365^®^) for descriptive statistical analyses. The differences were considered significant at a *p*-value less than 0.05 under chi-square tests using the GraphPad Prism v. 8 (Inc., San Diego, CA, USA).

## 3. Results

### 3.1. Morphological Identification and Description of Ixodes kashmiricus

Altogether, 352 *I. kashmiricus* ticks (Table 2) were collected in this study and morphologically identified. During this study, 94 out of 129 (72.87%) hosts of small ruminants including 56 sheep and 38 goats were infested by 352 ticks comprising adult females (175/352, 49.7%) followed by nymphs (115/352, 32.7%) and males (62/352, 17.6%). A significantly high prevalence of *I. kashmiricus* was found on sheep (271/352, 77%) followed by goats (81/352, 23%) in transhumant herds.

Furthermore, other tick species were not found co-infesting sheep and goats afflicted by *I. kashmiricus* ticks. During collection from district Chitral, only an adult female of *I. kashmiricus* was found on sheep. Details of host records, prevalence of ticks, and detection of *Theileria* and *Anaplasma* species in the selected districts are summarized in Table 2.

### 3.2. Sequences and Phylogenetic Relationship of Ticks

A sum of five ticks’ (one male, two adult females and two nymphs) genomic DNA was amplified via PCR targeting the *cox1* sequence. The BLAST analysis of the *cox1* sequence of *I. kashmiricus* showed 89.29% maximum identity with *I. scapularis* followed by 88.78% with *Ixodes gibbosus* and 88.71% with *Ixodes apronophorus* from Canada, Turkey and Russia, respectively. The obtained 16S rDNA sequence for *I. kashmiricus* was identical to the sequences of the same species from Pakistan (MW578839). Therefore, the 16S rDNA sequence was not included in further analysis. The obtained *cox1* sequence of *I. kashmiricus* was submitted to GenBank under the accession number OR244356.

**Figure 2:**
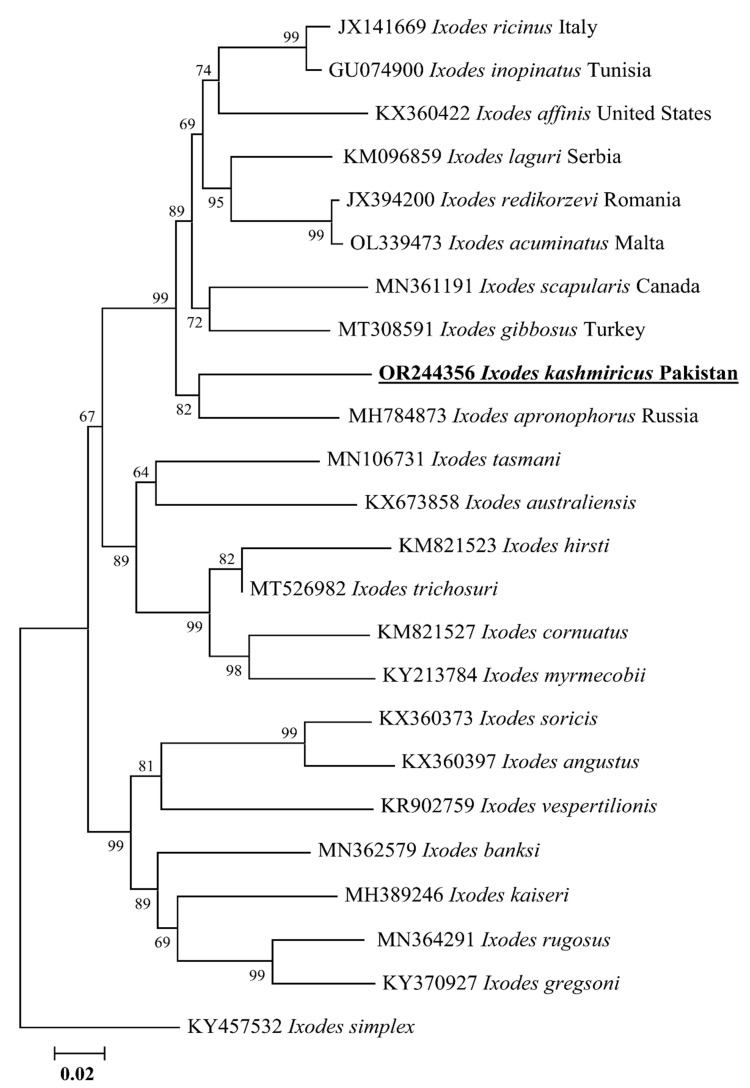
Phylogenetic tree of *Ixodes* species based on the *cox1* sequences. The *cox1* sequence of *Ixodes simplex* belonging to the subgenus *Eschatocephalus* was taken as an outgroup. The obtained *cox1* sequence was highlighted with bold and underlined fonts (OR244356).

Phylogenetically, the *cox1* sequence was clustered to the species of the subgenus *Ixodes ricinus* complex such as *I. apronophorus* (MH784873) reported from Russia. Furthermore, the *cox1* sequence formed sister clades with *I. ricinus* complex such as *I. scapularis*, *I. gibbosus, Ixodes acuminatus, Ixodes redikorzevi, Ixodes laguri, Ixodes inopinatus, Ixodes ricinus,* and *Ixodes affinis* reported from Canada, Turkey, Malta, Romania, Serbia, Tunisia, Italy and the United States (Figure 2).

### 3.3. Sequences and Phylogenetic Relationship of Theileria spp. and Anaplasma spp.

Among all molecularly analyzed ticks, *Theileria* spp. and *Anaplasma* spp. DNA were detected in two (1.65%: one adult female and one nymph) and four (3.3%: two adult females and two nymphs) *I. kashmiricus* ticks, respectively (Table 2). Moreover, other pathogens such as *Coxiella* spp. and *Borrelia* spp. based on *groEL* and *flaB* markers, respectively, were not amplified by PCR.

The 18S rDNA sequence of *Theileria* spp. showed 98.11% maximum identity with *Theileria* cf. *sinensis* reported from South Africa, which was followed by 97.99–97.87% identity with *T. sinensis* reported from Malaysia and China. Similarly, the 16S rDNA sequence of *Anaplasma* spp. showed 100% identity with *A. capra* reported from the Republic of Korea, China, and Iraq. The obtained 18S rDNA sequence of *T. sinensis*-like and 16S rDNA sequence of *A. capra* were submitted to GenBank (OR244360: *T. sinensis*-like and OR244358: *A. capra*). The details regarding the detection rate of *T. sinensis*-like and *Anaplasma capra* are shown in Table 2.

**Figure 3:**
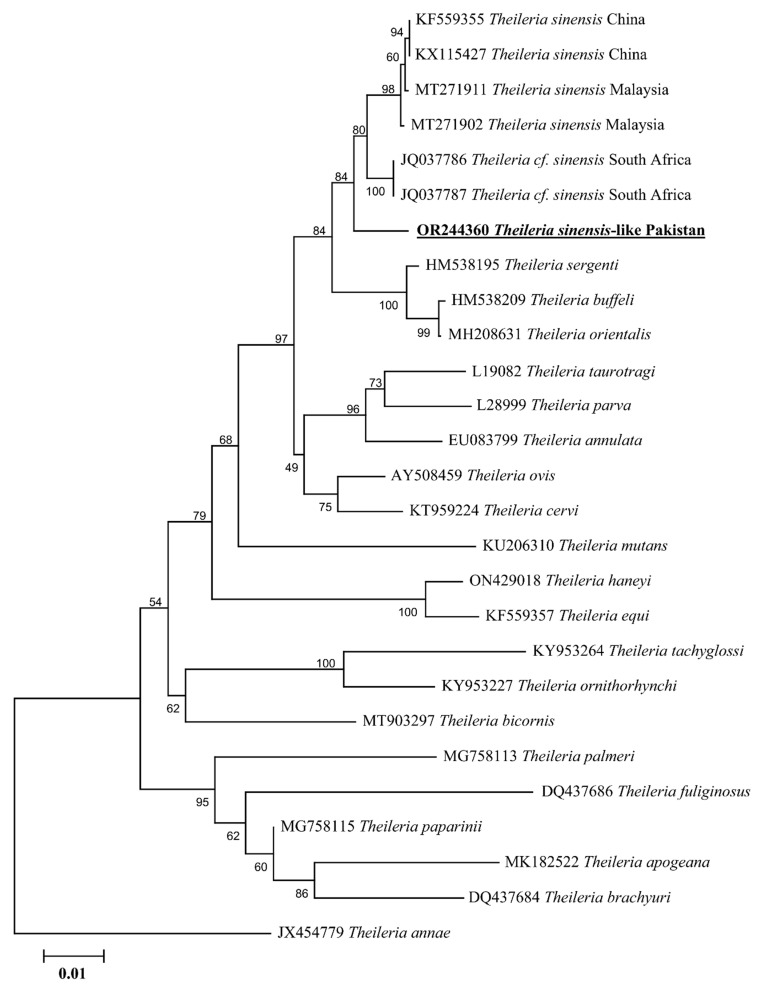
Phylogenetic tree of *Theileria* species based on the 18S rDNA sequences. The 18S rDNA sequence of *Theileria annae* was taken as an outgroup. The obtained 18S rDNA sequence was highlighted with bold and underlined fonts (OR244360).

**Figure 4:**
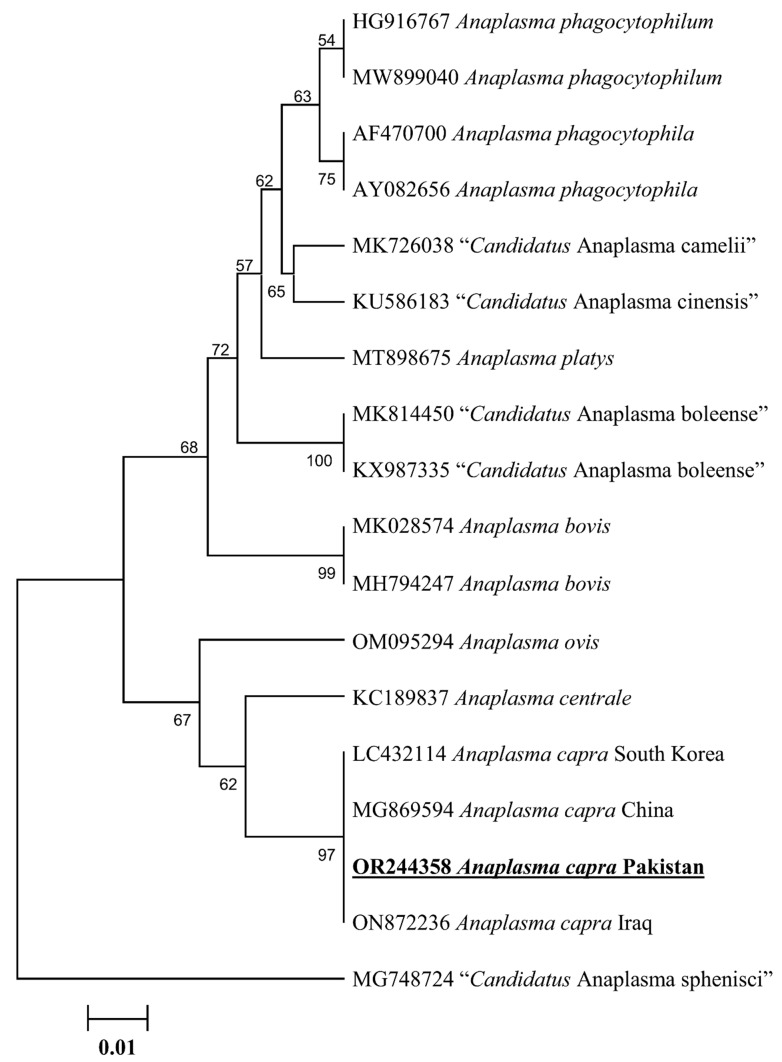
Phylogenetic tree of *Anaplasma* species based on the 16S rDNA sequences. The 16S rDNA sequence of “*Candidatus* Anaplasma sphenisci” was taken as an outgroup. The obtained 16S rDNA sequence was highlighted with bold and underlined fonts (OR244358).

The phylogenetic tree of the 18S rDNA sequence for *T. sinensis*-like clustered with *T. sinensis* (JQ037786-JQ037787) reported from South Africa and *T.* cf. *sinensis* reported from Malaysia (MT271902 and MT271911) and China (KX115427 and KF559355). It formed a sister clade with the sequences of *Theileria sergenti*, *Theileria buffeli* and *Theileria orientalis* (Figure 3). In the case of 16S rDNA, *A. capra* clustered to the corresponding species reported from South Korea (LC432114), China (MG869594), and Iraq (ON872236) (Figure 4).

## 4. Discussions

*Ixodes* ticks are known to transmit various pathogens such as *Anaplasma* spp., *Theileria* spp., *Coxiella* spp., *Rickettsia* spp., and *Borrelia* spp. to different hosts [5,7,11,14,20,21,42,43]. Genetic data of *I. kashmiricus* based on *cox1* sequence and their associated pathogens like *Theileria* spp. and *Anaplasma* spp. are missing. To date, a total of five *Ixodes* spp. such as *Ixodes hyatti* (Peshawar) [44], *Ixodes redikorzevi* (Kaghan) [45], *Ixodes stromi* [46], *I. kashmiricus* (Kashmir and Shangla) [21,33] and an undetermined *Ixodes* spp. (Swat) [47] have been reported in Pakistan. Among these, only *I. kashmiricus* (Shangla) has been characterized based on the morphology and molecular level [21]. In addition to the *Rickettsia* spp., *I. kashmiricus* associated with any other pathogens have not been characterized. In the current study, *I. kashmiricus* ticks collected from sheep and goats in district Shangla, Dir Upper and Chitral were characterized based on the *cox1* sequence and their associated *T. sinensis*-like and *A. capra* for the first time.

Small ruminants such as sheep and goats were found infested by *I. kashmiricus* ticks. The significantly higher infestation of *I. kashmiricus* on sheep among small ruminants shows that this tick prefers sheep as a host. The majority of the *Ixodes* spp. in the *I. ricinus* complex are associated with small ruminants: sheep and goats [33,48], which graze in areas having favorable climate conditions [49]. Similarly, the study districts are mountainous, having temperatures in the winter season below 10 °C, in the summer season 15–30 °C, a high relative humidity of ~70–80%, and precipitation throughout the year approximately 1000–1400 mm (climate-data.org, [26]). Notably, these transhumant herds seasonally migrate toward the frontiers of the country in northern and northwest areas during the spring and summer seasons (March–September). The frontiers of the country lie in the Palearctic region, which has a high prevalence and distribution of *Ixodes* ticks because of the availability of suitable environmental conditions [21,33,50]. Moreover, this transhumant movement of the infested hosts can enhance the dispersal of the *I. kashmiricus* ticks and associated pathogens to novel localities [51].

*Ixodes kashmiricus* ticks have been described previously by Pomerantzev [33] in India and then genetically characterized by Numan et al. [21] in Pakistan. These locations are at approximately 300 km (km) distance, while the current study’s new localities, Dir Upper and Chitral, are about ~130 km and ~165 km away from the previous collection site in the district Shangla, respectively. These ticks were collected from highly mountainous areas (up to 3000–3500 m elevation), as other members of the *I. ricinus* complex have been reported from hilly ranges in the Palearctic and Oriental regions [50]. Until the present study, only ITS and 16S rDNA sequences are freely available for *I. kashmiricus* in GenBank. Herein, we provided for the first time a cox1 sequence for *I. kashmiricus*, which shared a high identity with the *I. ricinus* complex. The morphological compatibility of *I. kashmiricus* was confirmed by molecular characterization, as the 16S rDNA sequence has close resemblance to the *I. ricinus* complex and clustered with the same species, which was previously reported in Pakistan. Whereas, due to the unavailability of *cox1* sequences for *I. kashmiricus* in GenBank, the obtained *cox1* sequence clustered to the *I. ricinus* complex from the Neotropical and Palearctic regions. The topologies of the constructed phylogeny for *I. kashmiricus* were paralleled to the sequences of *I. ricinus* complex [4,21].

Until the present study, except for the undetermined *Rickettsia* spp., no other pathogens in *I. kashmiricus* have been reported [21]. Ticks of the *I. ricinus* complex are the main vector of piroplasmids such as *T. annae*, *T. fuliginosa*, and undetermined *Theileria* spp. [14,17,18]. These *Ixodes*-associated pathogens have been genetically characterized based on the 18S rDNA. This genetic marker has been demonstrated to be valuable for determining evolutionary studies of protozoans [52,53,54,55]. The suggested identity or threshold level of 18S rDNA locus for *Theileria* spp. to be considered the same species is 99.3% [52]. However, the use of various parameters to determine genetic distances has led to insufficient use of this measure [18,55]. For instance, *Theileria fuliginosa* [18] and *Theileria ornithorhynchi* [56] have been considered similar species with 97.6% and 98.2% maximum identity, respectively. Likewise, the corresponding sequence of *T. sinensis*-like detected in *I. kashmiricus* showed 98.11% maximum identity. Furthermore, the obtained 18S rDNA sequence of *T. sinensis*-like showed a minimum nucleotide difference of 16 bp with the sequences of *T. sinensis*, which showed 1.89% (16/844 bp) genetic difference. Due to the high genetic differences, this species was considered as *T. sinensis*-like or related to *T. sinensis*. Similarly, the phylogeny of the obtained 18S rDNA sequence indicated a similar relationship or related to the *T. sinensis* reported from the Old World. The constructed phylogeny work has a comparable topology to those demonstrated by Loh et al. [18], whereas *Theileria* spp. is derived from *Ixodes* spp.

*Ixodes* ticks collected from cattle and sheep have been reported as a vector of *A. phagocytophilum* and *A. capra* [11,12,13,14,15]. This study presents the first evidence for *A. capra* in *I. kashmiricus*. For the molecular identification of *Anaplasma* spp., a highly conserved 16S rDNA sequence has been used historically [27,57]. Likewise, the 16S rDNA sequence of *A. capra* was detected in *I. kashmiricus*, which was reported for the first time. The present study reported *T. sinensis*-like and *A. capra* in *I. kashmiricus* ticks infesting small ruminants that closely related to the corresponding species. The zoonotic pathogenicity of the *T. sinensis*-like and *A. capra* was detected in this survey remains to be examined considering the significance of piroplasm and bacterial species as an agent of novel emerging infectious agents carried by *I. kashmiricus* ticks.

## 5. Conclusions

A new locality for *I. kashmiricus* was recorded, and its phylogenetic position based on the *cox1* sequence was delineated for the first time. Based on a phylogenetic analysis, the *I. kashmiricus* tick is closely related and clustered with the species of same subgenus—the *I. ricinus* complex. *Theileria sinensis*-like and *A. capra* were detected in *I. kashmiricus* for the first time. These findings may help to further understand the epidemiology of the *I. kashmiricus* tick and its associated *Theileria* and *Anaplasma* species, and they may strengthen the need for tick and tick-borne pathogen surveillance programs.

## Figures and Tables

**Table 1:** List of the primers used to amplify target DNA of the *Ixodes kashmiricus* and associated *Theileria* and *Anaplasma* species.

Organism/Marker	Primer Sequences 5′-3′	Amplicons	Annealing Temperature	References
Ticks/*cox1*	HC02198: TAAACTTCAGGGTGACCAAAAAATCA	649 bp	55 °C	[35]
	LCO1490: GGTCAACAAATCATAAAGATATTGG			
*Anaplasma* spp./16S rDNA	EHR16SD: GGTACCYACAGAAGAAGTCC	344 bp	55 °C	[36]
	EHR16SR: TAGCACTCATCGTTTACAGC			
*Theileria* spp./18S rRNA	18S_F: GGTAATTCTAGAGCTAATACATGAGC	897 bp	56 °C	This study
	18S_R: ACAATAAAGTAAAAAACAYTTCAAAG			
*Coxiella* spp./*groEL* *	CoxGrF1: TTTGAAAAYATGGGCGCKCAAATGGT	619 bp	56 °C	[37]
	CoxGrR2: CGRTCRCCAAARCCAGGTGC			
	CoxGrF2: GAAGTGGCTTCGCRTACWTCAGACG			
	CoxGrFR1: CCAAARCCAGGTGCTTTYAC			
*Borrelia* spp*./flab*	Fla SS: AAGAGCTGAAGAGCTTGGAATG	354 bp	55 °C	[38]
	Fla RS: CTTTGATCACTTATCATTCTAATAGC			

* Nested PCR.

**Table 2:** Prevalence of identified *Ixodes kashmiricus* ticks and their life stages and molecular detection of associated *Theileria* spp. and *Anaplasma* spp.

Location/ Districts	Host	Infested/Observed Hosts	Numbers of Ticks/Life Stages			Total Collected Ticks	*p* Value	Number of Ticks Subjected to DNA Isolation	Amplified *cox1* for *Ixodes kashmiricus*	Amplified 18S rDNA for *Theileria*	Amplified 16S rDNA for *Anaplasma*
			Males	Adult Females	Nymphs						
Shangla	Sheep	42/47	37	123	72	232	<0.001	77 (17M, 38F, 22N)	2 (1M, 1F)	1 (1F)	2 (1F, 1N)
	Goats	27/39	11	24	25	60		25 (13F, 12N)	1 (1F)	1 (1N)	1 (1N)
Dir Upper	Sheep	13/18	9	16	13	38		14 (3M, 6F, 5N)	1 (1N)	0	1 (1F)
	Goats	11/16	5	11	5	21		5 (3F, 2N)	1 (1N)	0	0
Chitral	Sheep	1/5	0	1	0	1		0	0	0	0
	Goats	0/4	0	0	0	0		0	0	0	0
Total Sheep (%)		56/70 (80)	46	140	85	271 (77)		91 (20M, 44F, 27N)	3 (1M, 1F, 1N)	1 (1F)	3 (2F, 1N)
Total Goats (%)		38/59 (64.4)	16	35	30	81 (23)		30 (16F, 14N)	2 (1F, 1N)	1 (1N)	1 (1N)
Overall Total (%)		94/129 (72.87)	62 (17.6)	175 (49.7)	115 (32.7)	352 (100)		121 (20M, 60F, 41N)	5 (1M, 2F, 2N)	2 (1.65) (1F, 1N)	4 (3.3) (2F, 2N)

## Data Availability

All the relevant data are within the manuscript.

## References

[1] Clifford C.M., Sonenshine D.E., Keirans J.E., Kohls G.M. (1973). Systematics of the subfamily Ixodinae (Acarina: Ixodidae). 1. the subgenera of *Ixodes*. Ann. Entomol. Soc. Am..

[2] Filippova N.A. (1977). Ixodid ticks of the subfamily Ixodinae. Fauna SSSR, Paukoobraznye, Izdatielstwo Nauka, Moskwa–Leningrad. Arachnida.

[3] Guglielmone A.A., Petney T.N., Robbins R.G. (2020). Ixodidae (Acari: Ixodoidea): Descriptions and redescriptions of all known species from 1758 to December 31, 2019. Zootaxa.

[4] Rar V., Yakimenko V., Tikunov A., Vinarskaya N., Tancev A., Babkin I., Epikhina T., Tikunova N. (2020). Genetic and morphological characterization of *Ixodes apronophorus* from Western Siberia, Russia. Ticks Tick Borne Dis..

[5] Pietzsch M.E., Medlock J.M., Jones L., Avenell D., Abbott J., Harding P., Leach S. (2005). Distribution of *Ixodes ricinus* in the British Isles: Investigation of historical records. Med. Vet. Entomol..

[6] Lommano E., Bertaiola L., Dupasquier C., Gern L. (2012). Infections and coinfections of questing *Ixodes ricinus* ticks by emerging zoonotic pathogens in Western Switzerland. Appl. Environ. Microbiol..

[7] Pilloux L., Baumgartner A., Jaton K., Lienhard R., Ackermann-Gäumann R., Beuret C., Greub G. (2019). Prevalence of *Anaplasma phagocytophilum* and *Coxiella burnetii* in *Ixodes ricinus* ticks in Switzerland: An underestimated epidemiologic risk. New Microbes New Infect..

[8] Sands B., Lihou K., Lait P., Wall R. (2022). Prevalence of *Babesia* spp. pathogens in the ticks *Dermacentor reticulatus* and *Ixodes ricinus* in the UK. Acta Trop..

[9] Guo W.P., Zhang B., Wang Y.H., Xu G., Wang X., Ni X., Zhou E.M. (2019). Molecular identification and characterization of *Anaplasma capra* and *Anaplasma platys*-like in *Rhipicephalus microplus* in Ankang, Northwest China. BMC Infect. Dis..

[10] Altay K., Erol U., Sahin O.F. (2022). The first molecular detection of *Anaplasma capra* in domestic ruminants in the central part of Turkey, with genetic diversity and genotyping of *Anaplasma capra*. Trop. Anim. Health Prod..

[11] Aktas M., Vatansever Z., Altay K., Aydin M.F., Dumanli N. (2010). Molecular evidence for *Anaplasma phagocytophilum* in *Ixodes ricinus* from Turkey. Trans. R. Soc. Trop. Med. Hyg..

[12] Bown K.J., Begon M., Bennett M., Woldehiwet Z., Ogden N.H. (2003). Seasonal dynamics of *Anaplasma phagocytophila* in a rodent-tick (*Ixodes trianguliceps*) system, United Kingdom. Emerg. Infect. Dis..

[13] Ogden N.H., Lindsay L.R., Hanincová K., Barker I.K., Bigras-Poulin M., Charron D.F., Heagy A., Francis C.M., O’Callaghan C.J., Schwartz I. (2008). Role of migratory birds in introduction and range expansion of *Ixodes scapularis* ticks and of *Borrelia burgdorferi* and *Anaplasma phagocytophilum* in Canada. Appl. Environ. Microbiol..

[14] Remesar S., Diaz P., Prieto A., García-Dios D., Panadero R., Fernández G., Brianti E., Díez-Baños P., Morrondo P., López C.M. (2021). Molecular detection and identification of piroplasms (*Babesia* spp.; *Theileria* spp.) and *Anaplasma phagocytophilum* in questing ticks from northwest Spain. Med. Vet. Entomol..

[15] Li H., Zheng Y.C., Ma L., Jia N., Jiang B.G., Jiang R.R., Huo Q.B., Wang Y.W., Liu H.B., Chu Y.L. (2015). Human infection with a novel tick-borne *Anaplasma* species in China: A surveillance study. Lancet Infect. Dis..

[16] Ali A., Ullah S., Numan M., Almutairi M., Alouffi A., Tanaka T. (2023). First report on tick-borne pathogens detected in ticks infesting stray dogs near butcher shops. Front. Vet. Sci..

[17] Camacho A.T., Pallas E., Gestal J.J., Guitián F.J., Olmeda A.S., Telford III S.R., Spielman A. (2003). *Ixodes hexagonus* is the main candidate as vector of *Theileria annae* in northwest Spain. Vet. Parasitol..

[18] Loh S.M., Paparini A., Ryan U., Irwin P., Oskam C. (2018). Identification of Theileria fuliginosa-like species in *Ixodes australiensis* ticks from western grey kangaroos (*Macropus fuliginosus*) in Western Australia. Ticks Tick Borne Dis..

[19] Wang Y., Wang B., Zhang Q., Li Y., Yang Z., Han S., Yuan G., Wang S., He H. (2021). The common occurrence of *Theileria ovis* in Tibetan Sheep and the first report of *Theileria sinensis* in yaks from southern Qinghai, China. Acta Parasitol..

[20] Sabitova Y., Rar V., Tikunov A., Yakimenko V., Korallo-Vinarskaya N., Livanova N., Tikunova N. (2023). Detection and genetic characterization of a putative novel *Borrelia genospecies* in *Ixodes apronophorus*/*Ixodes persulcatus*/*Ixodes trianguliceps* sympatric areas in Western Siberia. Ticks Tick Borne Dis..

[21] Numan M., Islam N., Adnan M., Zaman Safi S., Chitimia-Dobler L., Labruna M.B., Ali A. (2022). First genetic report of *Ixodes kashmiricus* and associated *Rickettsia* sp. *Parasit*. Vectors.

[22] Barker S.C., Walker A.R., Campelo D. (2014). A list of the 70 species of Australian ticks; diagnostic guides to and species accounts of *Ixodes holocyclus* (paralysis tick), *Ixodes cornuatus* (southern paralysis tick) and *Rhipicephalus australis* (Australian cattle tick); and consideration of the place of Australia in the evolution of ticks with comments on four controversial ideas. Int. J. Parasitol..

[23] Cheng T.Y., Chen Z., Li Z.B., Liu G.H. (2018). First report of *Ixodes nipponensis* infection in goats in China. Vector-Borne Zoonotic Dis..

[24] Lv J., Wu S., Zhang Y., Chen Y., Feng C., Yuan X., Jia G., Deng J., Wang C., Wang Q. (2014). Assessment of four DNA fragments (*COI*, 16S rDNA, ITS2, 12S rDNA) for species identification of the Ixodida (Acari: Ixodida). Parasit. Vectors.

[25] Ali A., Zahid H., Zeb I., Tufail M., Khan S., Haroon M., Bilal M., Hussain M., Alouffi A.S., Muñoz-Leal S. (2021). Risk factors associated with tick infestations on equids in Khyber Pakhtunkhwa, Pakistan, with notes on *Rickettsia massiliae* detection. Parasit. Vectors.

[26] Ali A., Numan M., Khan M., Aiman O., Muñoz-Leal S., Chitimia-Dobler L., Labruna M.B., Nijhof A.M. (2022). *Ornithodoros* (*Pavlovskyella*) ticks associated with a *Rickettsia* sp. in Pakistan. Parasit. Vectors.

[27] Khan Z., Shehla S., Alouffi A., Kashif Obaid M., Zeb Khan A., Almutairi M.M., Numan M., Aiman O., Alam S., Ullah S. (2022). Molecular survey and genetic characterization of *Anaplasma marginale* in ticks collected from livestock hosts in Pakistan. Animals.

[28] Ahmad I., Ullah S., Alouffi A., Almutairi M.M., Khan M., Numan M., Safi S.Z., Chitimia-Dobler L., Tanaka T., Ali A. (2022). Description of Male, Redescription of Female, Host Record, and Phylogenetic Position of *Haemaphysalis danieli*. Pathogens.

[29] Mangold A.J., Bargues M.D., Mas-Coma S. (1998). Mitochondrial 16S rDNA sequences and phylogenetic relationships of species of *Rhipicephalus* and other tick genera among Metastriata (Acari: Ixodidae). Parasitol. Res..

[30] Kovalev S.Y., Golovljova I.V., Mukhacheva T.A. (2016). Natural hybridization between *Ixodes ricinus* and *Ixodes persulcatus* ticks evidenced by molecular genetics methods. Ticks Tick Borne Dis..

[31] Karim S., Budachetri K., Mukherjee N., Williams J., Kausar A., Hassan M.J., Adamson S., Dowd S.E., Apanskevich D., Arijo A. (2017). A study of ticks and tick-borne livestock pathogens in Pakistan. PLoS Negl. Trop. Dis..

[32] Khan S.M., Khan M., Alouffi A., Almutairi M.M., Numan M., Ullah S., Obaid M.K., Islam Z.U., Ahmed H., Tanaka T. (2023). Phylogenetic Position of *Haemaphysalis kashmirensis* and *Haemaphysalis cornupunctata*, with Notes on *Rickettsia* spp. Genes.

[33] Pomerantzev B.I. (1948). New ticks of the family Ixodidae. Parazitol Sborn Zool Inst Akad Nauk. SSSR.

[34] Sambrook J., Fritsch E.F., Maniatis T. (1989). Molecular Cloning: A Laboratory Manual.

[35] Folmer O., Hoeh W.R., Black M.B., Vrijenhoek R.C. (1994). DNA primers for amplification of mitochondrial *cytochrome c oxidase* subunit I from diverse metazoan invertebrates. Mol. Mar. Biol. Biotech..

[36] Inokuma H., Raoult D., Brouqui P. (2000). Detection of *Ehrlichia platys* DNA in brown dog ticks (*Rhipicephalus sanguineus*) in Okinawa Island, Japan. J. Clin. Microbiol..

[37] Duron O., Noël V., McCoy K.D., Bonazzi M., Sidi-Boumedine K., Morel O., Vavre F., Zenner L., Jourdain E., Durand P. (2015). The recent evolution of a maternally-inherited endosymbiont of ticks led to the emergence of the Q fever pathogen, *Coxiella burnetii*. PLoS Pathog..

[38] Stromdahl E.Y., Williamson P.C., Kollars T.M., Evans S.R., Barry R.K., Vince M.A., Dobbs N.A. (2003). Evidence of *Borrelia lonestari* DNA in *Amblyomma americanum* (Acari: Ixodidae) removed from humans. J. Clin. Microbiol..

[39] Hall T., Biosciences I., Carlsbad C.J.G.B.B. (2011). BioEdit: An important software for molecular biology. GERF Bull Biosci..

[40] Kumar S., Stecher G., Li M., Knyaz C., Tamura K. (2018). MEGA X: Molecular evolutionary genetics analysis across computing platforms. Mol. Biol. Evol..

[41] Edgar R.C. (2004). MUSCLE: Multiple sequence alignment with high accuracy and high throughput. Nucleic Acids Res..

[42] Schouls L.M., Van De Pol I., Rijpkema S.G., Schot C.S. (1999). Detection and identification of *Ehrlichia*, *Borrelia burgdorferi* sensu lato, and *Bartonella* species in Dutch *Ixodes ricinus* ticks. J. Clin. Microbiol..

[43] Parola P., Paddock C.D., Socolovschi C., Labruna M.B., Mediannikov O., Kernif T., Abdad M.Y., Stenos J., Bitam I., Fournier P.E. (2013). Update on tick-borne rickettsioses around the world: A geographic approach. Clin. Microbiol. Rev..

[44] Clifford C.M., Hoogstraal H., Kohls G.M. (1971). *Ixodes hyatti*, n. sp.; and *I. shahi*, n. sp. (Acarina: Ixodidae), parasites of pikas (Lagomorpha: Ochotonidae) in the Himalayas of Nepal and West Pakistan. J. Med. Entomol..

[45] Begum F., Wisseman C.L., Casals J. (1970). Tick-borne viruses of west Pakistan: II. Hazara virus, a new agent isolated from *Ixodes redikorzevi* ticks from the Kaghan valley, w. Pakistan. Am. J. Epidemiol..

[46] Filippova N.A. (1957). *Ixodes stromi*, new species of tick and its systematic position in Ixodinae. Zool. Zhurnal..

[47] Begum F., Wisseman C.L., Traub R. (1970). Tick-borne viruses of West Pakistan: I. Isolation and general characteristics. Am. J. Epidemiol..

[48] Hoogstraal H. (1979). The epidemiology of tick-borne Crimean-Congo hemorrhagic fever in Asia, Europe, and Africa. J. Med. Entomol..

[49] Gilbert L., Aungier J., Tomkins J.L. (2014). Climate of origin affects tick (*Ixodes ricinus*) host-seeking behavior in response to temperature: Implications for resilience to climate change?. Ecol. Evol..

[50] Filippova N.A. (2002). Forms of sympatry and possible ways of microevolution of closely related species of the group *Ixodes ricinus-persulcatus* (Ixodidae). Acta Zool. Litu..

[51] Volkova V.V., Howey R., Savill N.J., Woolhouse M.E. (2010). Sheep movement networks and the transmission of infectious diseases. PLoS ONE.

[52] Schnittger L., Yin H., Gubbels M.J., Beyer D., Niemann S., Jongejan F., Ahmed J.S. (2003). Phylogeny of sheep and goat *Theileria* and *Babesia* parasites. Parasitol. Res..

[53] Schnittger L., Rodriguez A.E., Florin-Christensen M., Morrison D.A. (2012). *Babesia*: A world emerging. Infect. Genet. Evol..

[54] Sivakumar T., Hayashida K., Sugimoto C., Yokoyama N. (2014). Evolution and genetic diversity of *Theileria*. Infect. Genet. Evol..

[55] Ullah K., Numan M., Alouffi A., Almutairi M.M., Zahid H., Khan M., Islam Z.U., Kamil A., Safi S.Z., Ahmed H. (2022). Molecular Characterization and assessment of risk factors associated with *Theileria annulata* infection. Microorganisms.

[56] Paparini A., Macgregor J., Ryan U.M., Irwin P.J. (2015). First molecular characterization of *Theileria ornithorhynchi* Mackerras, 1959: Yet another challenge to the systematics of the piroplasms. Protist.

[57] Alam S., Khan M., Alouffi A., Almutairi M.M., Ullah S., Numan M., Islam N., Khan Z., Aiman O., Zaman Safi S. (2022). Spatio-temporal patterns of ticks and molecular survey of *Anaplasma marginale*, with notes on their phylogeny. Microorganisms.

